# Study on fluid inclusions and stable isotopics of W–Mo ore deposits in the Ningshan–Zhen’an area, South Qinling, China

**DOI:** 10.1038/s41598-024-67432-9

**Published:** 2024-07-16

**Authors:** Hujun He, Hongxu Tian, Ke Han, Xingke Yang, Yichen Zhao, Huixia Chao

**Affiliations:** 1 School of Earth Science and Resources, Chang’an University, Xi’an, 710054 China; 2 Key Laboratory of Western Mineral Resources and Geological Engineering, Ministry of Education, Chang’an University, Xi’an, 710054 China; 3 Xi’an Research Institute of China Coal Science Research Institute, Xi’an, 710000 China

**Keywords:** Fluid inclusion, H–O isotope, S isotope, W–Mo ore deposit, Metallogenic series, Mineralization area, South Qinling, Planetary science, Solid Earth sciences

## Abstract

The study of the fluid inclusions of W–Mo deposits in the mineralization area of Ningshan–Zhen’an , Shaanxi Province, China shows that the gas–liquid two-phase inclusions are mainly present in W–Mo deposits, and the ore-forming fluid can be divided into four types: high-temperature type, middle–high-temperature type, middle-temperature type and low-temperature type. The formation depths of the W–Mo mineralization range from 4.2 to 8.4 km. The boiling and mixing of fluid may have been important mechanisms for the formation of W–Mo mineralization. The skarn-type mineralization is dominated by magmatic water, the quartz-vein-type mineralization includes both magmatic water and meteoric water, and the meteoric water is more involved in the quartz-fluorite-vein-type, beryl-quartz-vein-type and pegmatite-type mineralization. Magma is the main source of sulfur; that is, magma is the main source of mineralization. Combined with the metallogenic setting and geological characteristics of typical ore deposits, in the process of structural system transformation in South Qinling, the ore-forming magma fluid in the Late Indosinian–Yanshanian period was uplifted and emplaced along the NW–WNW direction and NE–NNE direction, and eventually, NW–WNW fault-controlled skarn-type W–Mo mineralization and quartz-vein-type W–Mo deposits accompanied by greisenization, albitization and potash feldspathization formed.

## Introduction

**Figure 1:**
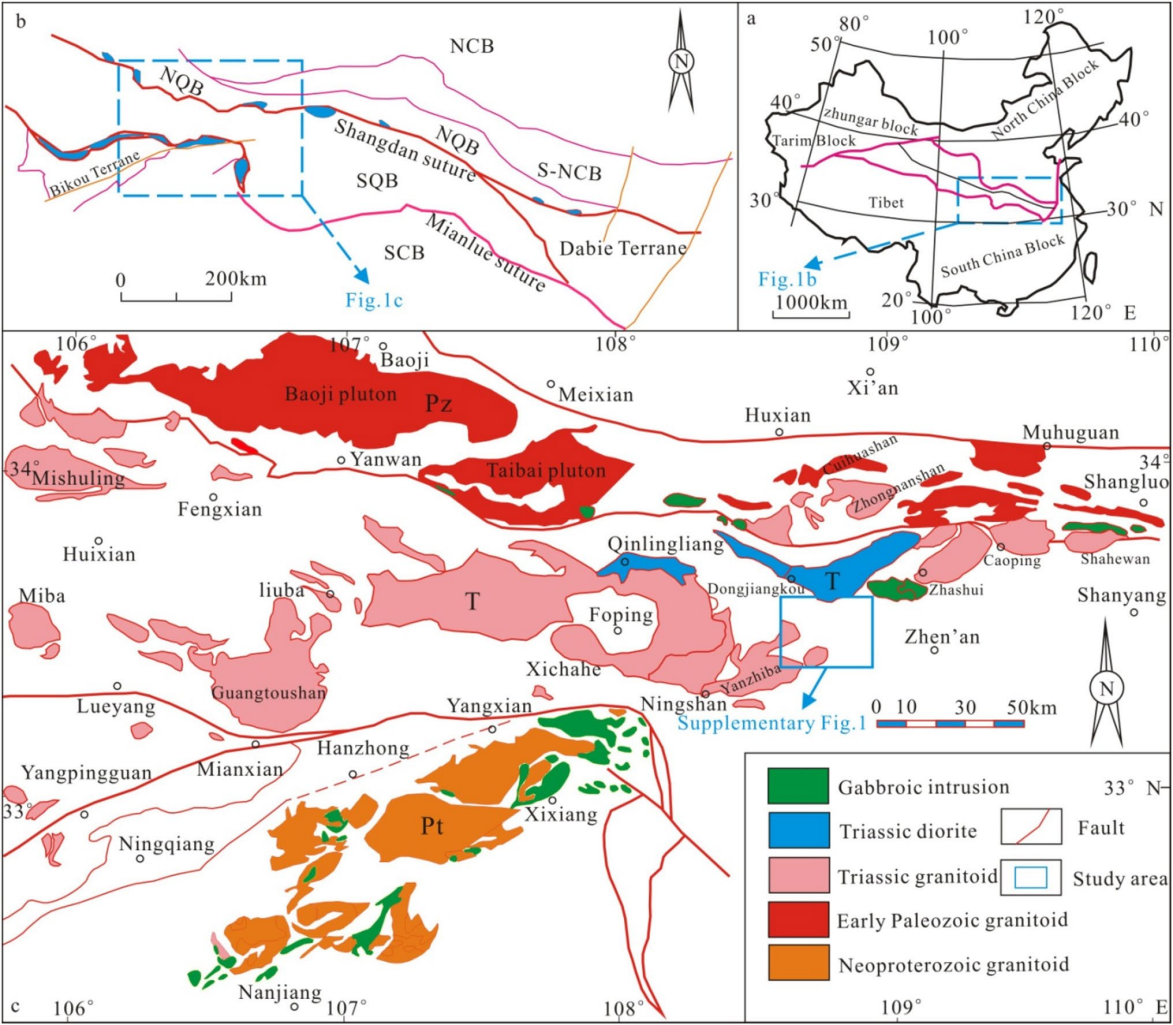
Simplified maps of (**a**) the location of the Qinling orogenic belt within China; (**b**) tectonic divisions of the Qinling orogenic belt; and (**c**) granite distribution in the South Qinling belt (modified after Dong et al.^28^). China basemap based on the China National Bureau of Surveying and Mapping Geographical Information. NCB, North China Block; S-NCB, southern margin of the North China Block; NQB, North Qinling belt; SQB, South Qinling belt; SCB, South China Block. The figure was generated using CorelDRAW X4 (https://www.corel.com/en/).

The Qinling orogenic belt, which lies in the hinterland of the Chinese mainland and structurally connects North China and the Yangtze Block, is a tectonic transition zone between the Paleo-Asian tectonic domain (or Altay series) and the Tethys tectonic domain (Fig. 1a)^1–3^. The unique structural position, more than 3.0 Ga of complex geological evolution, and the intense and special geological processes of the Qinling orogenic belt have resulted in abundant and special types of mineral resources. In particular, the Meso-Cenozoic period was the main ore-forming period, forming a world-class large–superlarge variety of tectono-magmatic fluids and sedimentary and metamorphic polymetallic mineral bases, such as Mo, Au, V, Pb, Zn, Ag, Hg, and U^3,4^. As an important part of the Qinling orogenic belt, South Qinling lies between the Mianlue and Shangdan suture belts, is an important metallogenic province in China and hosts several genetic types of deposits such as skarn- and quartz-vein-types W–Mo deposits, orogenic Au deposits etc^5–12^.

The W–Mo mineralization area in Ningshan–Zhen’an, Shaanxi Province, is located in the northern part of the Shangdan melange belt. It is an important part of the Shan–Zha–Zhen–Xun late Paleozoic–Mesozoic polymetallic metallogenic belt, South Qinling. In the mineralization area, various types of mineral resources have been found, including W, Mo, Au, Ag, Cu, Pb, Zn, Bi, Fe and V, at a total of approximately 126 sites. The dominant types of mineral resources are W, Mo, Cu, Pb, Zn, Au, and Ag. In recent years, the Shaanxi Geological Survey Center has carried out a number of metal-mineral investigations and research projects in the area and adjacent areas. Geochemical anomalies of Au, Mo, Pb, Zn, Ag, Cu, W, etc., have been found in the carbonate strata inside and around the Mesozoic intermediate–acidic large rock body (batholith) in the South Qinling area, and many W–Mo deposits (spots), such as Xinpu, Yueheping, Daxigou, Shentangou, Dongyang, Qipangou, Hetaoping, etc., have been found Moreover, the geological characteristics of the W–Mo deposits^13–15^, the geochemical characteristics of the deposits^16–19^, the ore-controlling structures^20–22^, the metallogenic epoch^19,23–25^, and the genesis of the deposits^13,16,17,19^ have been studied, providing a strong theoretical basis for subsequent exploration work in the mine area. However, the sources of ore-forming fluids and metals, as well as the metal enrichment processes in the mineralization area of Ningshan–Zhen’an, are still poorly understood. In this contribution, we present field observations, petrographic, microthermometric and Raman analyses of fluid inclusions combined with new stable H, O and S isotope studies of the Ningshan–Zhen’an W–Mo deposits to determine the origin of the ore-forming materials and evolutionary processes of the hydrothermal fluid system. These results can also provide significant insight into the mechanisms of large-scale ore precipitation, which could improve our understanding of ore genesis in the mineralization area of Ningshan–Zhen’an and other W–Mo deposits throughout the southern Qinling or Qinling orogenic belt.

## Regional geology

The mineralization area in Ningshan–Zhen’an is located in the tectonic area where the nearly E‒W tectonic belt of the Indosinian collision orogenic belt and the nearly N‒S tectonic belt of the Yanshanian intracontinental orogenic belt intersect in the South Qinling orogenic belt (Fig. 1b). The outcropping strata are mainly fine clastic–carbonate rocks of neritic shelf facies in the Devonian to upper Paleozoic strata, and there are local clastic rock and carbonate rock formations in the Sinian and Cambrian–Ordovician strata^26^. Faults, joints and fractures are well developed in the ore concentration area, and the strikes are generally WNW–WNW and NE–NNE. The combination of several major faults constitutes the “five vertical and three horizontal” tectonic framework, which plays a decisive role in the formation and occurrence of various mineral resources in the area. The intermediate–acidic magmatic rocks are well developed in the mineralization area and are mainly Mesozoic granites, which formed from 240 to 190 Ma and are Late Triassic–Early Jurassic in age, with a long time span. The rocks are mainly concentrated within 210–220 Ma and are Late Triassic in age. The last stage of magmatic activity was concentrated within 200–190 Ma and occurred in four rock bodies (Dongjiangkou, Yanzhiba, Lanbandeng and Shihaiping) in the Early Jurassic. The four main intrusions are characterized by composite rock body and petrological facies changes. The long duration of magmatic activity and the younger age of the rock body indicate the occurrence of multiple stages of magmatic intrusion, which may also be accompanied by multiple episodes of mineralization or multiple stages of the same stage of magmatic intrusion^19,24–29^. The Xiaomoling complex is distributed in the northeastern part of the mineralization area, followed by the Hercynian diorite intrusive rock. In addition, a large number of granitic dikes have developed in the area; they vary in width and can reach several meters. They are mainly distributed along the NE–NNE strike in rock masses and surrounding rocks. Pegmatite dikes are distributed in a network of veins along several groups of joints, which is consistent with the structural occurrence of the late Indosinian–Yanshanian; most of these dikes are closely related to mineralization (Fig. 1c)^27^. The mineralization area is dominated by regional metamorphic rocks, which have the most extensive distribution, while the dynamic metamorphic rocks are closely associated with the faults and folds in the area. The contact metamorphic rock is mainly located in a particular area of the contact zone between the intermediate–acidic magmatic rock and the carbonate or clastic rocks.

The favorable ore-forming stratigraphic and structural magmatic rock conditions in the mineralization area of Ningshan–Zhen’an inevitably led to the formation of a large amount of metal mineralization. More than 10 kinds of metallic minerals have been found in the ore concentration area (Supplementary Fig. 1), including Mo, W, Au, Cu, Pb, Zn and V. Of these, the reserves of W, Mo, V, Au and Pb, Zn are the most significant; a number of medium-large W–Mo deposits and V deposits have been found, and the number of small-scale deposits and mineralization points has reached hundreds, mainly W–Mo deposits in Dongyang and Guilingou in Zhen’an and Mo deposits in Shentangou and Fujiagou, Ningshan.

## Samples and analytical methods

### Fluid inclusion analyses

The samples for fluid inclusion analyses are collected from ore-bearing quartz veins, mineralized skarns, and ore-bearing pegmatite dikes in the mineralization area (Supplementary Table 1). Fluid inclusion petrography, microthermometry and Raman probe analysis are studied from (how much thick) μm thick double-sided thin sections.

The petrography, microthermometry and laser Raman analysis of the fluid inclusions were conducted at the Key Laboratory of Western Mineral Resources and Geological Engineering, Ministry of Education, Chang’an University. The use of instruments and the detailed test, analysis and calculation processes are detailed in the references^17,30–33^.

### H–O isotope analyses

A total of 11 quartz-bearing ore samples were selected for H–O isotope testing of the quartz inclusions. The samples were crushed to 40–80 mesh, and the pure quartz minerals were selected under the microscope; their purity was greater than 99%. The testing of H–O isotopes of quartz and S isotopes of sulfides was carried out at the Beijing Research Institute of Uranium Geology. The use of instruments and the detailed test process are detailed in the references^34^.

### S-isotope analyses

In this work, 9 ore samples from the Qipangou, Yueheping, Guilingou, Daxigou, and Yanggou-Di’ergou mine areas were first crushed to 40–80 mesh, after which molybdenite and pyrite with purities greater than 95% were selected under a binocular microscope and then ground to 200 mesh for sulfur isotope analysis. Sulfur isotope testing was performed at the Beijing Research Institute of Uranium Geology. The use of instruments and the detailed test process are detailed in the references^34,35^.

## Results

### Characteristics of fluid inclusions

#### Petrographic characteristics of fluid inclusions

The fluid inclusions in the minerals, which are mainly ellipsoids, are well developed (Supplementary Table 2), and their sizes were 5–20 μm. According to Lu et al.^36^, the fluid inclusions in the Ningshan–Zhen’an area can be classified into vapor–liquid phases (type I), pure-liquid phases (type II) and pure-vapor phases (type III) (Supplementary Fig. 3).

Vapor–liquid two-phase (type I): This type is composed of a vapor phase and aqueous solution and mostly develops in fluorite and quartz mineral crystals. This type of inclusion can be further divided into a liquid-rich phase (type I-a, with more than 70% liquid phase) and a vapor-rich phase (type I-b). Type I-a comprises elliptically shaped fluid inclusions up to 21 μm in diameter, primarily distributed in a linear and isolated pattern within quartz crystals, with the volume fraction of the vapor phase from 90 to 92%(Supplementary Fig. 3a–c). The vapor–liquid ratio of type I-b inclusions is high (more than 40%), and they have relatively low abundances of quartz, fluorite and scheelite. The color of the inclusions are generally vivid, and the inclusions are generally large, with particle sizes of 10–20 μm and elliptical shapes (Supplementary Fig. 3d and e).

Pure-liquid phase (type II): Single-phase inclusions are distributed in quartz and fluorite, mainly in scattered or isolated form, which are associated with the above types of inclusions, but the number of inclusions is small and elliptical. Type II inclusions are usually colorless and transparent (Supplementary Fig. 3d).

Pure-vapor phase (type III): Type III inclusions are generally grayish black and difficult to observe (Supplementary Fig. 3b and c). In addition to the above types, CO_2_-bearing and subcrystalline polyphase inclusions are rare (Supplementary Fig. 3f.).

#### Characteristics of the homogenization temperature and salinity of fluid inclusions

The freezing-point temperature and homogenization temperature of the inclusions were obtained by the freezing method and homogenization method, respectively.

Based on the statistical results of the homogenization temperature in the mineralization area and the research results of Liu^13^ on fluid inclusions in the Dongyang mine area, the ore-forming fluids of Mo and W mineralization in the mineralization area can be classified into four types (Supplementary Table 2 and Supplementary Fig. 4): (1) high-temperature type (peak values of 355–380 °C), (2) middle–high-temperature type (209–327 °C), featuring Mo–W mineralization of the quartz-vein type in other mine areas; (3) middle-temperature type (19–213 °C), featuring Mo mineralization of the feldspar-quartz-pegmatite type; and (4) low-temperature type (154–189 °C), featuring the W mineralization quartz–fluorite-vein type and the W mineralization beryl–quartz-vein type.

#### Density characteristics of fluid inclusions

Based on the density formula of vapor–liquid two-phase inclusions^33,35,37,38^, the statistical results of the fluid inclusion density in the mineralization area are shown in Supplementary Table 2. The densities of the ore-forming fluids of quartz-vein-type, pegmatite-type, quartz-fluorite-vein-type, etc., in other mine areas are similar, except for the densities of the quartz-vein-type and skarn-type ore-forming fluids in the Qipangou mine area, which are relatively small.

#### Capture pressure and depth

The capture pressure of the fluid inclusions in the quartz veins was calculated, and the capture depth was estimated based on the empirical formula^39,40^ and a Microsoft Excel spreadsheet based on the *PVTX* (pressure–volume–temperature–composition) properties of H_2_O–NaCl (Steele-MacInnis et al.)^41^ of fluid pressure. According to the estimated fluid-capture pressure-metallogenic depths (Supplementary Table 2), the formation depths of the skarn-type and quartz-vein-type W mineralization in the Qipangou mine area are the greatest, ranging from 8.09 to 8.44 km. The depths of the Mo–W mineralization of the quartz- vein-type in other mine areas are 6.08–7.77 km, which are slightly greater than that of the Qitangou mine area. The depths of the pegmatite-type mineralization are 5.09–5.82 km, which is greater than that of the quartz-vein-type mineralization. The quartz-fluorite-vein-type mineralization and beryl-quartz-vein-type mineralization are located in the shallowest parts of the mineralization area at depths of approximately 4.23–5.48 km. The vertical zonation at the different mineralization depths is similar to the distribution characteristics of the homogenization temperature and salinity above.

#### Laser Raman analysis of inclusions

The results of the laser-Raman microprobe analysis show that the vapor-phase compositions of the quartz inclusions in the ore-bearing quartz veins are mainly CO_2_ and CH_4_, while the contents of N_2_, SO_2_ and H_2_O are not significant; H_2_O is the main component in the liquid phase (Supplementary Fig. 5). In the skarn-type mineralization, there is a single vapor component in the quartz inclusions, mainly CO_2_, and the liquid component is mainly H_2_O. The vapor components of the pegmatite-type mineralization are mainly CO_2_ and SO_2_, which is consistent with Liu's research^13^ on Dongyang W deposits.

### Stable isotopes

#### H–O isotopes

The H–O isotope analysis results for the quartz inclusions are shown in Supplementary Table 3.

**Figure 2:**
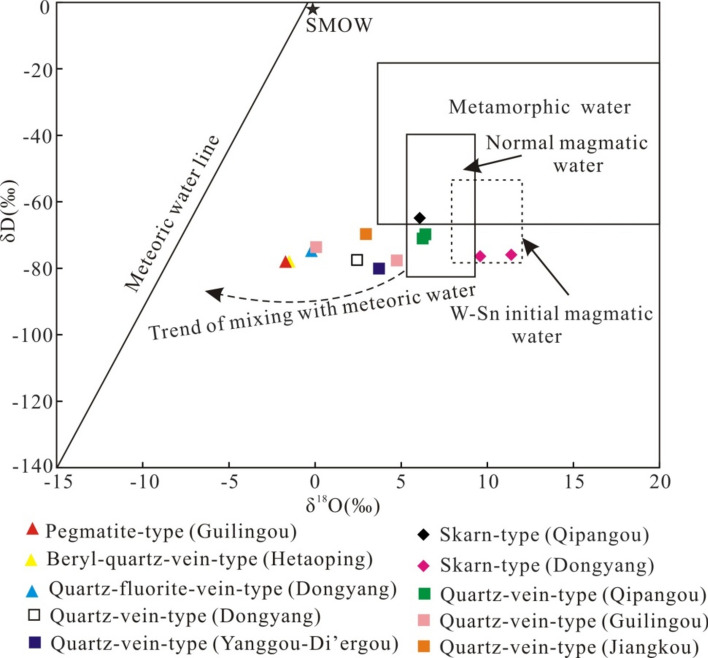
Diagram of δ^18^O_H2O_-δD isotopic-fluid properties in the mineralization area. The base map is based on Taylor^44^, and the initial magmatic water of the W–Sn series is based on Zhang^45^.

The test results of the quartz in the different W–Mo mineralization types in the mineralization area show that the δD values range from − 64.9 to − 80.1‰ and that the mean value is − 74.4‰, which is within the range of normal magmatic water. The δ^18^O values of the quartz in the different W–Mo mineralization types in the mineralization area range from 7.3 to 13.8‰, and the mean value is 11.4‰, which is consistent with the δ^18^O values of crust-remelting granites (10.0–12.0‰)^42^. According to the quartz–water equilibrium fractionation Equation ^43^, the final δ^18^O_H2O_ values are − 1.71–6.42‰, and the mean value is 2.67‰. Overall, the δD values vary little due to the small effect of water–rock interactions on the δD values, and the H isotopes of the different ore-forming types are similar; however, from skarn-type mineralization to quartz-vein-type mineralization, followed by beryl-quartz-vein-type, quartz-fluorite-vein-type and pegmatite-type mineralization, the O isotopes decrease in turn. On the H–O isotope diagrams, all the samples in the Qipangou mine area are in the magmatic water field, and the skarn-type mineralization in the Dongyang mine area plots within the initial magmatic-water range of the W–Sn series. The other samples are all located between the magmatic-water range and the meteoric water line. The quartz-fluorite-vein-type, beryl-quartz-vein-type and pegmatite-type mineralization continue to drift to the meteoric water line, indicating that magmatic water was the main component of the ore-forming fluid in the skarn-type mineralization stage. During the hydrothermal-vein mineralization stage, the hydrothermal fluid gradually mixed with meteoric water. In the late stages of quartz-fluorite-vein-type, beryl-quartz-vein-type and pegmatite-type mineralization, meteoric water was more involved in mineralization (Fig. 2).

#### S isotopes

The results of the analysis of the S isotopes are shown in Supplementary Table 4.

**Figure 3:**
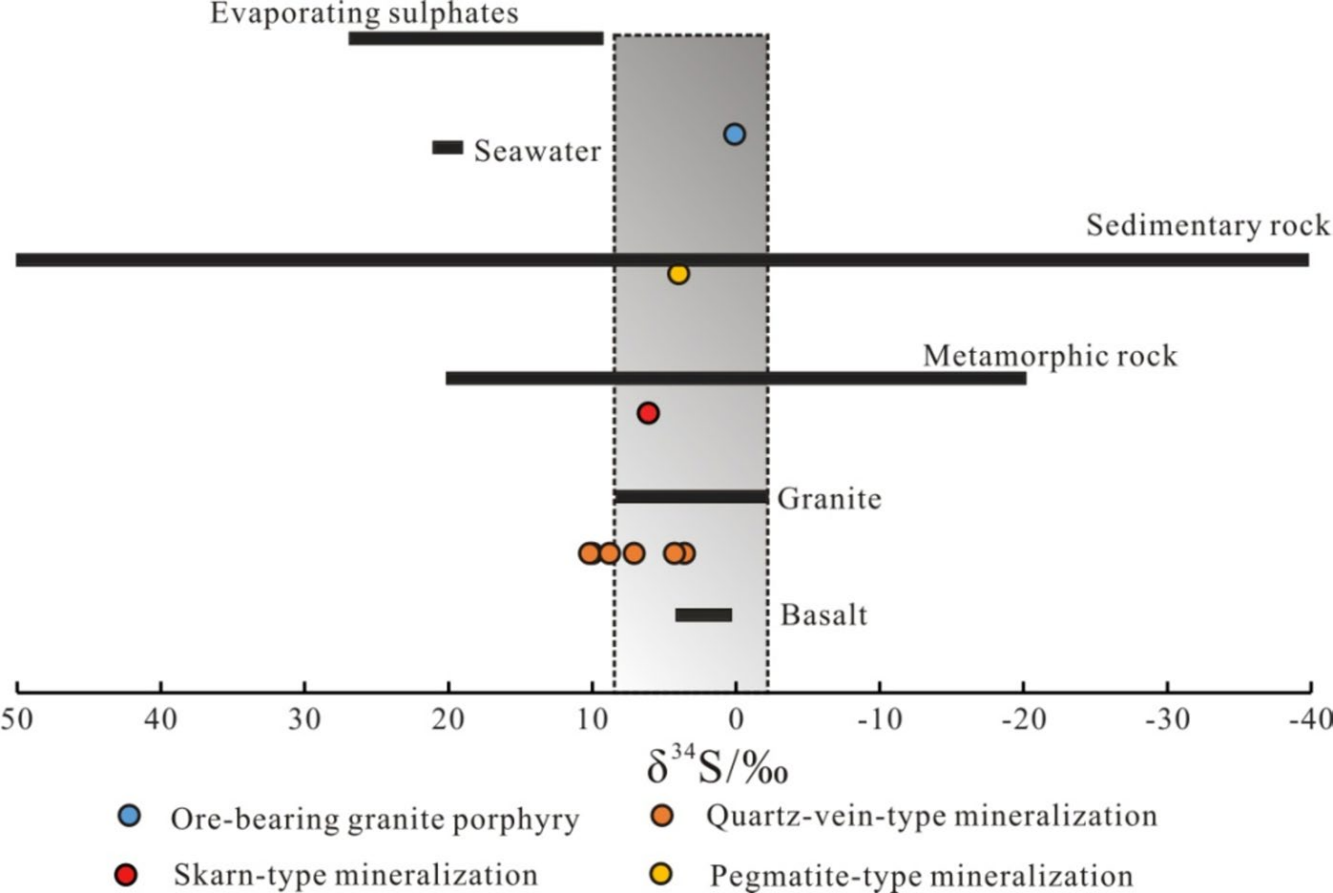
Distribution of the S-isotopic composition of W–Mo deposits in the mineralization area of Ningshan–Zhen’an. The base map is based on Hoefs^47^.

The test results showed the following. The δ^34^S values of the ore-bearing quartz veins are 3.6–10.2‰, with a mean value of 7.3‰. The δ^34^S value of the skarn-type mineralization is 6.1‰, the δ^34^S value of the pegmatite-type mineralization is 4‰, and the δ^34^S value of the granite-porphyry-type Mo deposit of Daxigou is 0.1‰. All the samples demonstrate positive values and have magmatic sulfur features (Fig. 3). The δ^34^S of the granite–porphyry Mo deposit of Daxigou is nearly 0, which indicates that it is a deep-source sulfur deposit. The δ^34^S values of quartz-vein-type, skarn-type and pegmatite-type mineralization range from 3.6 to 10.2‰, which are essentially within the δ^34^S range of granitic magma (5–15‰; Fig. 3)^46^. The above data indicate that sulfur may mainly arise from deep magmas and may be contaminated by surrounding rocks, which indicates that the S isotopes are gradually homogenized through crust–mantle interactions during the metallogenic process; the ore-forming fluid may have arisen from a unified fluid system of reservoir–crust–mantle interaction processes, and the ore-forming material in the ore concentration area may have mainly arisen from granite (acidic igneous rock).

## Discussion

### Nature and evolution of the fluids

Based on the statistical results of the homogenization temperature and salinity of the fluid inclusions, the fluid salinity of the quartz-vein-type inclusions in the Dongyang mine area is greater than that of the pegmatite-type and quartz-fluorite-vein-type inclusions. In the Qipangou mine area, the fluid salinities of the skarn-type and quartz-vein-type are similar, and the span is large. In the Hetaoping mine area, the fluid salinity of the quartz-vein-type is greater than that of the beryl quartz-vein-type. In the Yanggou-Di’ergou mine area, the fluid salinity of the quartz-vein-type is generally greater than that of the pegmatite type and the quartz fluorite type. The fluid salinity in the mineralization area generally shows the same distribution characteristics as the homogenization temperature; namely, the quartz-vein-type and skarn-type ore-forming fluids generally exhibit intermediate–high temperatures and moderate–high salinities. The pegmatite type, quartz-fluorite-vein type and beryl-quartz-vein type show the characteristics of middle–low-temperature and middle–low-salinity fluids.

The quartz, fluorite and scheelite crystals of different types of mineralization in the mineralization area feature the coexistence of various types of inclusions with similar homogenization temperatures, and fluid boiling may have occurred^36^; this is also one of the main reasons for the mineralization of W, Mo and other metals^38^. The skarn-type Mo–W deposits and quartz-vein-type Mo–W deposits are metallogenic fluids with intermediate–high temperatures and high salinities and may boil at 355–380 °C, resulting in the precipitation of early W–Mo deposits. The fluid upwells and migrates continuously along the tectonic belt, the physical and chemical conditions of the system change, and W–Mo mineralization continues. The lower homogenization temperature and salinity of the ore-forming fluids in the late stage may have decreased due to the mixing of fluids, resulting in the precipitation of quartz, fluorite and beryl in the late stage, accompanied by W–Mo mineralization. In conclusion, fluid boiling and mixing may be reasons for the formation of W–Mo mineralization in the mineralization area.

Higher contents of H_2_, CO, CH_4_ and C_2_H_4_ in the fluid may represent a stronger reducibility of the fluid, indicating a deeper source environment of the fluid, and vice versa for a weaker reducibility, representing a shallower formation environment of the fluid. The presence of N_2_ in the vapor phase of fluid inclusions indicates that ore-forming fluids may have formed by the mixing of multiple origins fluids^48,49^. The compositions of quartz-bearing fluid inclusions with different mineralization types or stages in the mineralization area were analyzed and compared. There was more CH_4_ and less CO_2_ in the quartz-fluid inclusions of the quartz-vein-type W mineralization in the Qipangou mine area (Supplementary Fig. 5c); the results show that the fluid was mainly reductive, which is favorable for the transport of W. There was little or no CH_4_ and less CO_2_ in the skarn-type mineralization, which may have been related to the number of samples detected (only one sample was detected in skarn-type mineralization). In the quartz-bearing fluid inclusions from the Dongyang, Hetaoping and Yanggou-Di’ergou mine areas, the CH_4_ content obviously decreased, while the CO_2_ content increased; moreover, there was little SO_2_ and N_2_ in some of these areas, indicating that the fluid reducibility decreased and slightly deviated from oxidation and that other source fluids may have been added. Pegmatite-type mineralization does not contain CH_4_, and in vapor-phase compositions, CO_2_ and SO_2_ are common; therefore, it is also a partially oxidized fluid. The results of the analysis of the redox properties of the fluid inclusions are consistent with those of the above studies on temperature, salinity and estimated depth, which indicate the metallogenic environment. The quartz-vein-type ore-forming fluid in the Qipangou mine area are medium–high-temperature and medium–high-salinity; the mineralization occurred in the reducing deep environment of the mineralization areas. The quartz-vein-type ore-forming fluid in the Dongyang, Hetaoping and Yanggou-Di’ergou mine areas are medium-temperature and medium–low-salinity, which formed in a reduction–partial-oxidation environment in the shallow part of the mineralization area. The pegmatite-type ore-forming fluid had low temperatures and salinities; it formed in the shallow oxidizing environment of the mineralization area.

### Source of ore-forming fluid and mechanism of mineral precipitation

According to the research results of the fluid inclusions in the mineralization area, both magmatic water and meteoric water are present in the ore-forming fluid and may have boiled and mixed. The redox environments of the different mineralization types are also different. If there is only one stage of mineralization in the mineralization area, the early stage may have been a partially reduced environment, which gradually changed to a reducing–partially oxidizing environment with the upwelling evolution of the ore-forming fluid along the tectonic belt. Based on the geological characteristics of the surface outcrop and the existence of blind granite bodies in the depths of the mineralization area and the results of the S isotope measurements, it can be inferred that the ore-forming fluid in the mineralization area was mainly derived from hydrothermal magma in the deep areas. During the process of magmatic hydrothermal fluid ascending and migrating along the tectonic belt, the temperature gradually decreased, after which the magmatic hydrothermal fluid mixed with meteoric water in the later stage. The mineralization mechanism of W, Mo and other metals may involve the boiling and mixing of fluids during the process of upward fluid migration.

The δ^34^**S** values can be used to broadly identify three different S sources: (1) mantle-derived (or magmatic) S has δ^34^**S** values of 0 ± 3‰, (2) seawater-derived sulfur has δ^34^**S** values of ~  + 20‰, and (3) sedimentary S typically has highly variable but negative δ^34^S values due to the isotopic fractionation caused by bacteria and redox processes. Additionally, (4) mixed S can contain contributions from the three aforementioned sources and has variable δ^34^S values^47,50^. The S isotopic compositions of the typical deposits in the mineralization area are relatively concentrated, and the δ^34^S values are basically positive, indicating that the sulfur is mainly magmatic (Fig. 3). The results show that the positive δ^34^S values of the metal sulfides are mainly related to magmatic degassing or marine sulfates. Magmatic SO_2_ degassing can lead to a significant loss of ^34^S in the sulfides of rocks, and the δ^34^S value can change from 0 to − 8‰. Magmatic H_2_S degassing can lead to a relative enrichment of ^34^S in the sulfides of rocks, and the δ^34^S value can change from 0 to 6‰^43^. Therefore, the positive δ^34^S values of the W–Mo mineralization area in Ningshany–Zhen'an may be the result of magmatic degassing. SO_2_ is generally detected in the vapor phase of fluid inclusions (Supplementary Fig. 5). SO_2_ and H_2_S are two common S-containing gases in magmatic volatiles, while ^34^S is more enriched in SO_2_ than in H_2_S and melts; therefore, large amounts of H_2_S escaping from a magmatic system can lead to an increase in the δ^34^S values (> 0‰) of sulfides in melts or late ore-forming hydrothermal solutions^51^. Magmatic volatiles also tend to have higher N_2_ contents^52^. The discovery of N_2_ in the inclusions of the W–Mo mineralized quartz veins also suggests that magmatic fluids may be involved in mineralization.

At temperatures of 200–300 °C, NaWO_4_^−^ and HWO_4_^–^ are the main migration forms of W, while at 400–600 °C, NaHWO_4_ is the main migration form^53,54^. The metallogenic temperatures of W and Mo in the W–Mo mineralization area in Ningshan–Zhen’an are mostly in the 300 °C range; therefore, NaWO_4_^-^ and HWO_4_^-^ may be the main forms of W in the ore-forming fluid in the mineralization area.

The vapor phase of fluid inclusions in mineralization areas generally contains CO_2_ gas. Although a debate is underway as to whether the presence of CO_2_ in fluids facilitates the migration and enrichment of W and Mo^55,56^, changes in the acidity and alkalinity of melt or ore-forming hydrothermal fluids and increases in metal concentrations are associated with CO_2_ loss^57^. Other studies have shown that the loss of CO_2_ can reduce the solubility of W in fluids, leading to precipitation and mineralization^58^. As mentioned above, typical fluid immiscibility or fluid boiling signs were observed in the quartz vein-mineralized fluid inclusions in the mineralization areas, and it was found that one of the causes of fluid boiling may be the mixing of different fluids^59^. Thus, the original equilibrium state of the fluid has been destroyed. Wood and Samson^60^ and Redkin and Kostromin^61^ have shown that changes in the temperature, Ph, Eh, etc., of ore-forming fluids have a significant effect on the stability of W–Mo complexes. When these physical and chemical factors change in the ore-forming fluid system, the complex of W and Mo easily decomposes, which leads to the enrichment, precipitation and mineralization of W and Mo. The temperature range of the ore-forming fluid in the mineralization area is large (between 100 and 500 °C). The main ore-forming temperatures in the mineralization area are between 200 and 300 °C and lower than 350 and 500 °C, except that the ore-forming temperature in the Qipangou mine area is generally higher than 300 °C, which is the general mineralization temperature of W and Mo^60^. This indicates that one of the main causes of W and Mo mineralization is fluid mixing and that the effect of cooling is less significant.

According to the metallogenic setting and typical geological characteristics of the W–Mo mineralization area in Ningshan–Zhen’an , during the transformation of the tectonic system in South Qinling, the tectonic–magmatic activities in the mineralization areas were intense; the extensional rock-controlling and ore-controlling fault systems of different scales developed widely and caused the late Indosinian–Yanshanian granitic magma to intrude into the lower part of the mineralization area, which created favorable conditions for the formation and migration of ore-forming fluids. This provided a structural pathway for the mixing of meteoric water seeping down to a certain depth in the Earth's crust and ore-forming hydrothermal fluid. In the ore-forming hydrothermal system after mixing, due to abrupt changes in physical chemistry, the physical chemistry system of the fluid balance was disrupted, and large amounts of ore-forming material began to accumulate and precipitate.

## Conclusions

1. The W–Mo mineralization in the mineralization area of Ningshan–Zhen’an is mainly skarn- and quartz-vein-types and is controlled by NW–WNW and NE–NNE faults, joints, fissures, etc. W–Mo mineralization is usually located in the contact zone between the granitic body and carbonate rocks.
2. The ore-forming fluid related to the quartz-vein-type W deposits in the Qipangou mine area is middle–high-temperature and middle–high-salinity fluid, which formed in the deep environment of the mineralization area, with partially reducing conditions. The quartz-vein-type ore-forming fluid in the Dongyang, Hetaoping and Yanggou-Di’ergou mine areas is intermediate-temperature and low-salinity; it formed in the shallowly reducing and partially oxidizing environment of the mineralization area. The pegmatite-type mineralized ore-forming fluid is low in temperature and salinity; it formed in a shallow, partially oxidizing environment. The depths of the Mo–W mineralization are 4.2 –8.4 km.
3. The skarn-type mineralization is mainly composed of magmatic water. Both magmatic water and meteoric water are present in quartz-vein-type mineralization, and in quartz-fluorite vein-type, beryl-quartz vein-type and pegmatite-type mineralization, meteoric water is more heavily enriched in magma and is the main source of sulfur; that is, magma is the main source of mineralization.

### Supplementary Information

Supplementary Information.

## Data Availability

All the data generated or analyzed during this study are included in the paper and its Supplementary Information files.

## References

[1] Huang JQ, Chen BW (1987). The Evolution of the Tethys in China and Adjacent Regions.

[2] Chen YJ (2010). Indosinian tectonic setting, magmatism and metallogenesis in Qinling Orogen, central China. Geol.. China.

[3] Zhang GW, Zhang BR, Yuan XC, Xiao QH (2001). Qinling Orogenic Belt and Continental Dynamics.

[4] Zhang X, Shi LC, Cheng SS, Duan CY, Wei YQ, Deng DW, Lu YY (2019). Aeromagnetic characteristics and fractue structure framework of the eastern part of the western Qinling orogen. Geol China.

[5] Wang PA, Chen YC (1997). Tectono-minerogenic cycles and minerogenetic evolution through geological history in the Qinling orogenic belt. J. Geomech..

[6] Wang JH, Zhang FX, Yu ZP, Yu L (2002). Minerogenetic series of metallic ore deposits in the Qinling Mountains and tectonodynamic background of the continental orogenic belts. Geol. China.

[7] Yao SZ, Ding ZJ, Zhou ZG, Chen SY (2002). Metallogenic system of Qinling orogen. Earth Sci. J. China Univ. Geosci..

[8] Du YL, Tang ZL, Cai KQ, Li WY, Zhang T (2003). Relationship between Indosinian–Yanshanian tectonic framework and large-superlarge mineral deposits in Qinling–Qilian Orogenic belt. Miner. Depos..

[9] Mao JW, Xie GQ, Zhang ZH, Li XF, Wang YT, Zhang CQ, Li YF (2005). Mesozoic large-scale metallogenic pulses in North China and corresponding geodynamic settings. Acta Petrol. Sin..

[10] Zhu LM, Zhang GW, Li B, Guo B, Yao AP, Gong HJ (2009). Some key metallogenic events of Qinling orogenic belt and their deposit examples. J. Northwest Univ..

[11] Yan Z, Wang ZQ, Chen L, Liu SW, Ren T, Xu XY, Wang RT (2014). Tectono-magmatism and metallogeneses of Shanyang–Zhashui ore concentration area in Qinling Orogen. Acta Petrol. Sin..

[12] Zhang ZY, Wang YH, Liu JJ, Zhang FF (2020). Geology, fluid inclusions, and H–O–S–Pb isotopes of the Chigou porphyry Cu deposit in Southern Qinling, central China: Implication for ore genesis. Ore Geol. Rev..

[13] Liu X (2013). The characteristics and Genesis of W Deposit in Zhen'an, Shaanxi province.

[14] Liu B, Li L (2020). Geological characteristics and metallogenic conditions of Dongyang tungsten deposit, Zhen'an County, Shaanxi Province. World Nonferr. Metals.

[15] Zhang WS (2020). Geological Characteristics of Tungsten–Molybdenum Ore Field and Study on the Relationship between Hidden Rock Masses and Mineralization in Western Zhen'an, South Qinlin.

[16] Wang JM, Cao HY, Dong SQ, Zhu M, Shi LY, Dang C, You J (2017). Genetic discussion and geological and characteristics of geochemical of Jinpen tungsten deposit in Zhen'an, Shaanxi province. Sci. Technol. Eng..

[17] Ruan SQ (2019). Tectonic and Fluid Characteristics and Genesis Model of Qipangou Tungsten Deposit in West Zhen’an South Qinling.

[18] Ruan SQ, Yang XK, Zhu W, Gao YF, Han K (2019). Study on characteristics of ore-forming fluids in Qipangou Tungsten mining area, Western of Zhen’an, Shaanxi. Gold Sci. Technol..

[19] Han K (2021). Ore-controlling Structure-Magma-Fluid-Metallogenetic Regularity and Prospecting Prediction in the W-Mo-Au Polymetallic ore Concentration Area in Ningshan–Zhen’an Area.

[20] Gao YF (2019). Characteristics of Ore-Controlling Structure and Metallogenic Model of Typical Tungsten Deposits in Western Zhen'an, South Qinling, South Qinling.

[21] Gao YF, Yang XK, Ruan SQ, Han K, Zhang WS, Zhu W (2019). Characteristics of ore-controlling structures and prospecting indicators in the Western Tungsten deposit area of Zhen’an, South Qinling. Gold Science and Technology.

[22] Yang XK, He HJ, Chao HX, Han K, Liu XW, Zhu W, Wei L, Jia FY (2021). Discovery and significance of deep prospection of Late Indosinian–Yanshanian intracontinental orogenic overpass structure magmatic fluid superposition mineralization in Ningshan–Zhen’an tungsten molybdenum gold field in South Qinling. Geol. Shaanxi.

[23] Zhang W, Wang GB, Zhang K, Wang JL, Hu Y, Wang PP, Wang F, Liu WJ, Chen Y, Chen YW, Zhou XL, Ji CK (2020). Indosinian ore-forming process of the Qipangou W deposit in Zhen'an, Shaanxi: Evidence from hydrothermal zircon U-Pb dating. Geol. China.

[24] Han K, Yang XK, Chao HX, He HJ, Ruan SQ, Gao YF, Zhang WS, Zhu W, Jin G (2021). U-Pb Zircon and Re-Os Molybdenite Geochronology of The W-Mo Mineralized Region of South Qinling, China, and Tectonic Implications. Acta Geol. Sin..

[25] Chao HX, Cui WW, Yang XK, Wang R, Han K, Yang N, Wang YZ (2023). Phlogopite ^40^Ar–^39^Ar Dating of the Qipangou Tungsten deposit in Western Zhen’an, South Qinling mountains and its significance of late Indosinian ore mineralization. Geotectonica et Metallogenia.

[26] Shaanxi Institute of Geological Survey (2017). Regional geology of China-Shaanxi.

[27] Du YE, Ji CK (2017). Discussion on metallogenic regularity and prospecting potential of molybdenum-tungsten deposits in the west section of Yuehe, Zhen’an county. West-China Explor. Eng..

[28] Dong YP, Liu XM, Zhang GW, Chen Q, Zhang XN, Li W, Yang C (2012). Triassic diorites and granitoids in the Foping area: Constraints on the conversion from subduction to collision in the Qinling orogeny, China. J. Asian Earth Sci..

[29] Yang XK, Chao HX, He HJ (2018). Research Report on Magmatism and W-Mo Mineralization in the Mineral Concentrated Area of Western Zhen’an.

[30] Brown PE (1989). FLINCOK: A microcomputer program for the reduction and investigation of fluid-inclusion data. Am. Mineral..

[31] Bodnar RJ (1993). Revised equation and table for determining the freezing point depression of H_2_O-NaCl solutions. Geochim. Cosmochim. Acts.

[32] Hall DL, Sterner SM, Bodnar RJ (1988). Freezing point depression of NaCI-KCl-H_2_O solutions. Econ. Geol..

[33] Liu B (2001). Density and isochoric formulae for NaCl-H_2_O inclusions with medium and high salinity and their applications. Geol. Rev..

[34] Zhu YD (2011). Magmatic characteristics and genesis of porphyry Mo (Cu) deposit in the Tongcun of Zhejiang Province.

[35] Yang JJ, Yang XK, Yang CD, Li Q, Yang FQ (2022). Genesis of the Talate Pb–Zn (–Fe) deposit in the Altay, Xinjiang, NW China: Evidence from fluid inclusions and stable isotopes. Ore Geol. Rev..

[36] Lu, H. Z., Fan, H. D., Ni, P., Ou, G. X., Shen, K. & Zhang, W. H. *Fluid Inclusion.* Science Press

[37] Liu ZH, Luo DZ, Qi D, Liu H, Gao D, Wu X (2015). Period and fluid inclusions geochemistey of quartz veins in Jiangjunhe area, Shiquan county, shaanxi province. Northwest. Geol..

[38] Kang M, Wang LY, Zhu XF, Chen Y, Liu XF, Yue CC (2017). Study on fluid inclusions of the Xishanwanyangchang silver ore deposit in volcanic rocks, Inner Mongolia. Acta Petrol. Sin..

[39] Shao JL, Mei JM (1986). Study on typomorphic characteristics of mineral inclusion in the gold deposit from volcanic terrain in Zhejiang and its genetic and prospecting significance. Miner. Rocks.

[40] Liu B, Shen K (1999). Thermodynamics of Fluid Inclusion.

[41] Steele-MacInnis M, Lecumberri-Sanchez P, Bodnar RJ (2012). HOKIEFLINCS_H2O-NACL: A Microsoft Excel spreadsheet for interpreting microthermometric data from fluid inclusions based on the *PVTX* properties of H_2_O-NaCl. Comput. Geosci..

[42] Chen YX, Zheng YF, Chen RX, Zhang SB, Li QL, Dai MN, Chen L (2011). Metamorphic growth and recrystallization of zircons in extremely ^18^O-depleted rocks during eclogite-facies metamorphism: Evidence from U-Pb ages, trace elements, and O–Hf isotopes. Geochim. Cosmochim. Acta.

[43] Clayton RN, O'Neil JR, Mayeda TK (1972). Oxygen isotope exchange between quartz and water. J. Geophys. Res..

[44] Taylor HP (1974). The application of oxygen and hydrogen isotope studies to problems of hydrothermal alteration and ore deposition. Econ. Geol..

[45] Zhang LG (1987). Oxygen isotope studies of wolframite in tungsten ore deposits of South China. Geochimica.

[46] Ohmoto H, Goldhaber MB, Barnes HL (1997). Sulfur and carbon isotopes. Geochemistry of Hydrothermal Ore Deposits.

[47] Hoefs J (2015). Stable Isotope Geochemistry.

[48] Gao YB, Li WY, Zhang ZW (2011). Fluid inclusions and H–O isotopic compositions of quartz-vein ores in the Baiganhu–Jialesai W–Sn mineralization belts, Qimantage, NW China. Acta Petrol. Sin..

[49] Luo XP, Xue CJ (2011). Characteristics of fluid inclusions and stable isotope composition of Chahansala gold deposit, Western Tianshan, Xinjiang, China. Acta Geol. Sin..

[50] Huang C, Li XF, Wang LF, Liu FP (2013). Fluid inclusion study of the Huangshaping polymetallic deposit, Hunan Province, South China. Acta Petrol. Sin..

[51] Zhao J (2017). Mineralogical study and in situ sulfur isotopic characteristics of Au-carrying pyrites in Shuiyindong gold deposit, Guizhou Province.

[52] Zheng YF, Fu B, Zhang XH (1996). Effects of magma degassing on the carbon and sulfur isotope compositions of igneous rocks. Sci. Geol. Sin..

[53] Giggenbach, W. F. *Composition of magmatic components in hydrothermal fluids.* Short Course Handbook (1995).

[54] Liu YJ, Ma DS (1987). Geochemistry of Tungsten.

[55] Ma DS (2009). Progress in research on geochemistry of tungsten. Geol. J. China Univ..

[56] Wang XD, Ni P, Jiang SY, Huang JB, Sun LQ (2008). Fluid inclusion study on the Piaotang tungsten deposit, southern Jiangxi province, China. Acta Petrol. Sin..

[57] Cao XF, Lv XB, He MC, Niu H, Du BF, Mei W (2009). An infrared microscope investigation of fluid inclusions in coexisting quartz and wolframite: A case study of Yaogangxian quartz.vein wolframite deposit. Miner. Depos..

[58] Liu XC, Zhang DH (2019). The efficient mechanisms for precipitating wolframite: CO_2_ escaping. J. Geomech..

[59] Zhang ZL, Huang ZL, Rao B, Wen B, Yan ZF (2005). Study on the ore-forming fluid characteristics of huize Pb–Zn ore deposits. Contrib. Geol. Miner. Resour. Res..

[60] Wood SA, Samson IM (2000). The hydrothermal geochemistry of tungsten in granitoid environments: I. Relative solubilities of ferberite and scheelite as a function of T, P, pH, and mNaCl. Econ. Geol..

[61] Redkin AF, Kostromin NP (2010). On the problem of transport species of tungsten by hydrothermal solutions. Geochem. Int..

