# Supplementary material for: Study on fluid inclusions and stable isotopics of W–Mo ore deposits in the Ningshan–Zhen’an area, South Qinling, China
Source: Sci Rep. 2024 Jul 16;14:16440. doi: 10.1038/s41598-024-67432-9 (PMC11252120; doi:10.1038/s41598-024-67432-9)
Supplement: Supplementary file 1 — Supplementary Information. [file 41598_2024_67432_MOESM1_ESM.docx]

**Supplementary material**

**Study on fluid inclusions and stable isotopics of W-Mo ore deposits in the Ningshan-Zhen'an area, South Qinling, China**

Hujun He^1,2,*^, Hongxu Tian^1^, Ke Han^3^, Xingke Yang^1^, Yichen Zhao^1^, Huixia Chao^1^

1. School of Earth Science and Resources, Chang’an University, Xi’an 710054, China

2. Key Laboratory of Western Mineral Resources and Geological Engineering, Ministry of Education, Chang’an University, Xi’an 710054, China

3. Xi'an Research Institute of China Coal Science Research Institute, Xi’an 710000, China

*Correspondence should be addressed to Hujun He; hsj2010@chd.edu.cn

**Deposits geology**

The W-Mo mineralization area in Ningshan-Zhen’an, Shaanxi Province, is located in the northern South Qinling tectonic belt. The W deposits are mainly distributed in the Shiwengzi Formation and Liangchakou Formation. These W-Mo deposits (mineralized) have a close spatial relationship with the magmatic rocks in the mineralization area (the mineralized area is located in the contact zone between the rock mass and the wall rock or in the rock mass) (Supplementary Fig. 1). The quartz-vein-type and skarn-type W-Mo deposits are hosted by NW–NWW and NE–NNE faults in Cambrian–Ordovician clastic and carbonate rocks^19,24-29^.


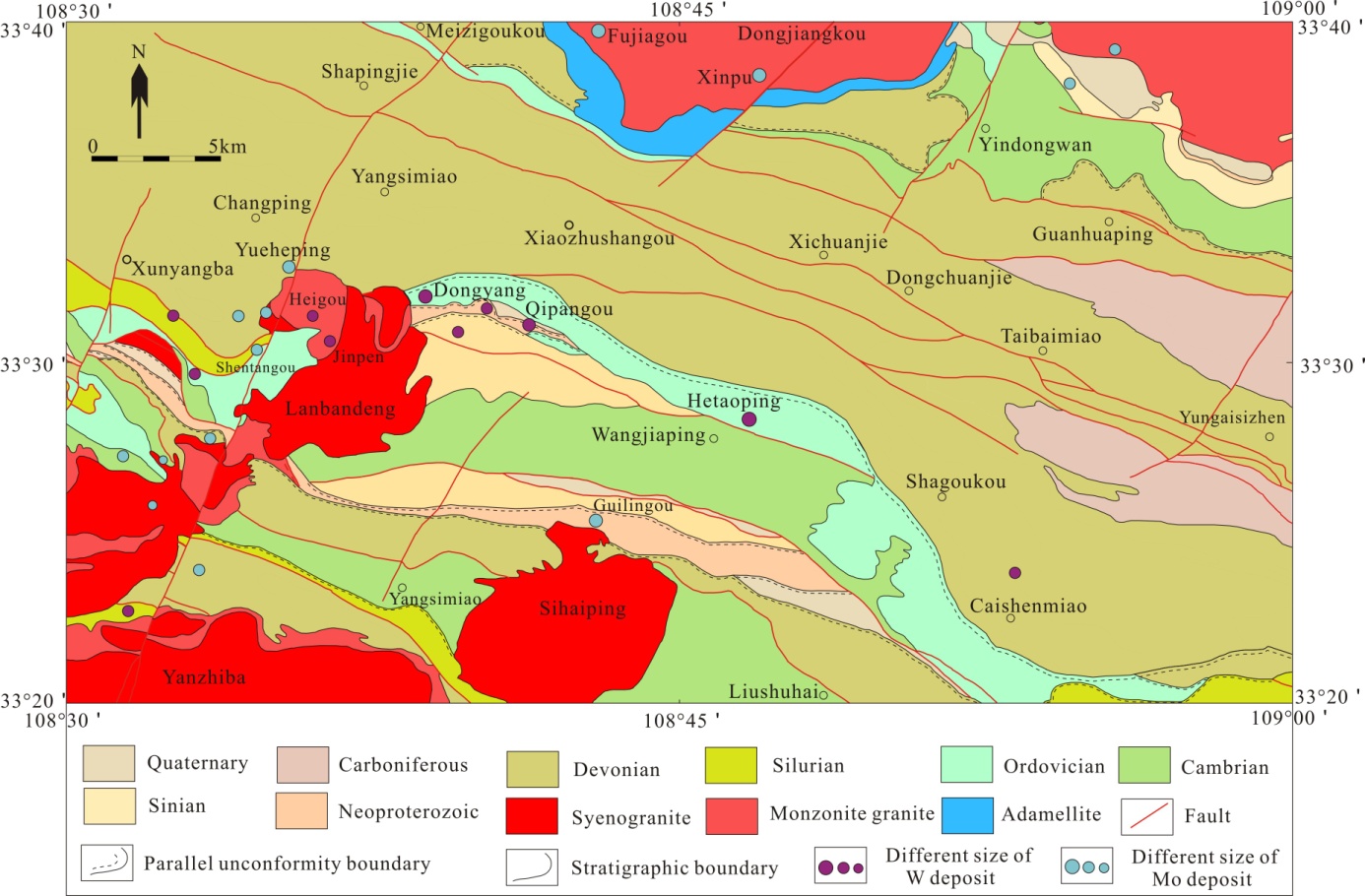


**Supplementary Figure 1.** Simplified geological map of the W-Mo mineralization area of South Qinling (modified according to Yang et al., 2018)^29^. The figure was generated using CorelDRAW X4 (https://www.corel.com/en/).


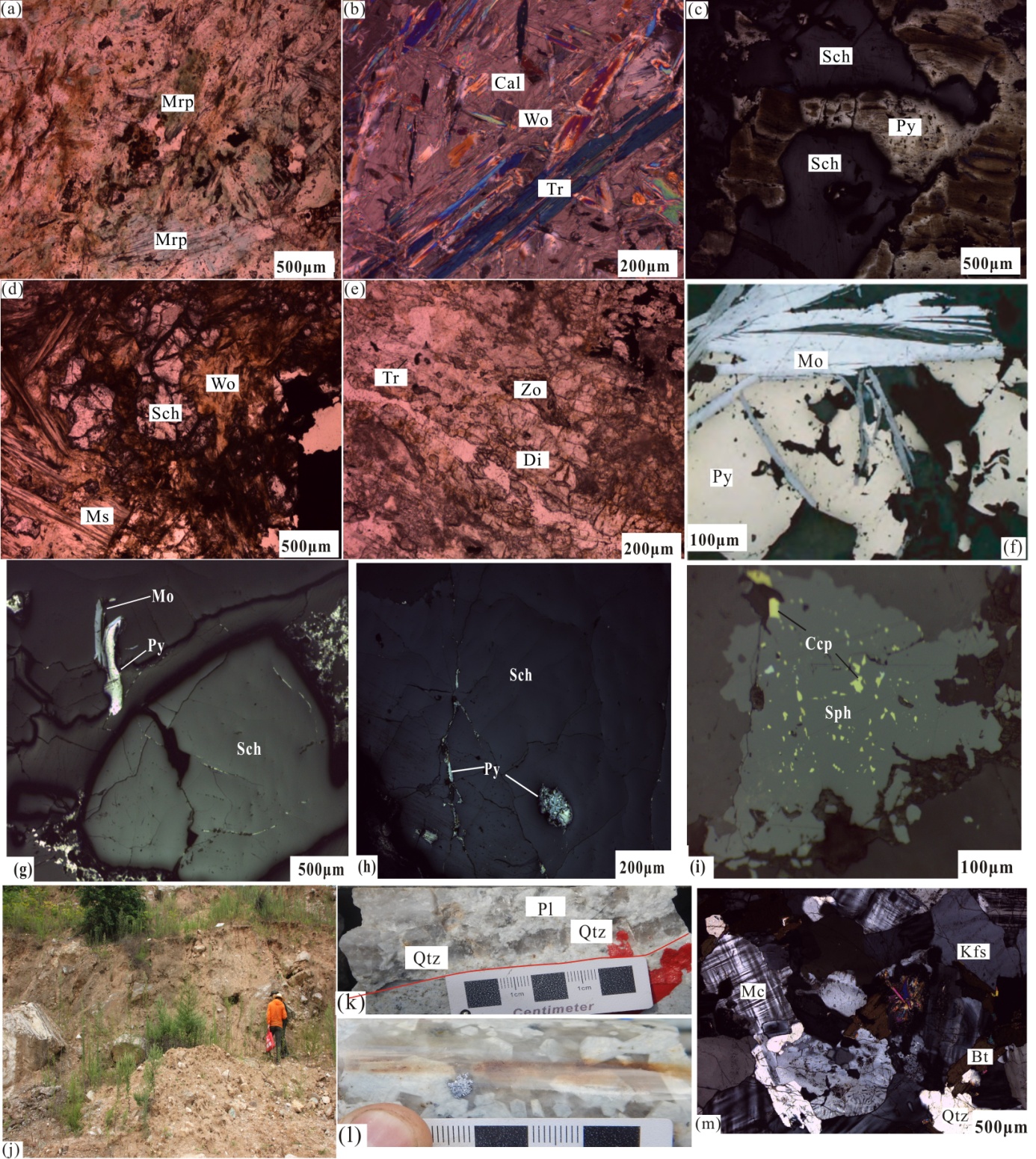


**Supplementary Figure 2.** Mineralization characteristics in the mineralization area of Ningshan-Zhen’an. a, b, c, d, e, Microstructural characteristics of skarn-type ore; f, microstructural characteristics of Mo ore; g, microstructural characteristics of quartz-vein-type scheelite; h, scheelite fracture filled with pyrite; i, scheelite fracture filled with pyrite; j, residual diluvial-type W orebody; k, feldspar–quartz pegmatite vein; l, star–dot-shaped Mo ore in pegmatite vein; m, microstructural characteristics of porphyritic biotite monzonitic granite. Brl. beryl, Bt. biotite, Cal. calcite, Ccp. chalcopyrite, Di. diopside, Kfs. K-feldspar, Mc. microcline, Mo. molybdenite, Mrp. mariposite, Pl. plagioclase, Py. pyrite, Qtz. quartz, Wo. wollastonite, Sch. scheelite, Sph. sphalerite, Tr. [tremolite](https://wenda.so.com/q/1604112757217569), Wf. wolframite, Zo. zoisite.

Skarn-type W-Mo orebodies occur in stratigraphic rocks of different ages around intermediate–acidic intrusive rock bodies. They are mainly present in carbonate rocks, such as limestone, dolomite, and marble, and in various types of Paleozoic strata. The limestone, marble, and skarn marble outcrops have different widths, ranging from several meters to several tens of meters. The main skarn types are calcareous skarns, magnesite skarns, tremolite diopside skarns, pyrite garnet skarns, epidote [actinolite](https://www.so.com/link?m=bZXNRPxXiXO8CLPLHHERve%2FHp4LHqyBhaV9nAyLK7aHEuwrxEvmlDCh2NWYhOqXf62FC3RVpIvzDTB7OHS7oMUM3WQ493Bv%2FGx6wUewe%2BBQsSpnurr2me0E8aRJ6c2dFED1RWcta8Pov8fjNhUaCNbzG06rR8TcB2cQIxScOfiDkQ%2FtGdhTR2Ni6LfyBnEp2Mru8EmlZN2JI2hAEO) skarns, epidote diopside skarns, muscovite epidote diopside skarns, etc. There are dozens of known skarn (mineralization) belts in the area, which are 100~1000 m in length and 20~120 m in width. They are layered or [stratoid](https://www.so.com/link?m=bTwAn59McG9kmFUGBBUoo7nGTJigMTp0IM9%2FF7tF5khKEF5eo9nnOaosozBynWeKod2fvc%2FEGUv%2BYlSarREDU8vDHMHfoxSy%2FvG2EOVFMk1A6%2FuYnmx8hNs07E8xiC2D6ks4Sb0GJ3KfnxfRtaNtgwyIR9uMcW%2B66C5Sw%2FFYMn9jbpXjQUyIWjZIl%2BOb87JqRrn5XGQ%3D%3D). The skarn-type W-Mo orebodies are 900~1300 m long and 1.97~5.1 m thick. The strikes are nearly E‒W–WNW, the dips are N–NNE, and the grades of WO_3_ are 0.42%~1.16%. The orebody is controlled by the fracture zone near the E‒W–W direction. The main skarn minerals are wollastonite, diopside, tremolite, garnet, muscovite, biotite, phlogopite, beryl, etc. Mo-W ore and Pb-Zn ore have been found in some skarns (Supplementary Figs. 3a, b, c, d, e).

The quartz vein W-Mo orebodies are 43~570 m long and 0.46~1.09 m thick, the strikes are nearly N–N–NNE, and the WO_3_ grades are 0.7~1.09%. The orebodies are predominantly controlled by the NE–NNE and nearly N‒S faults or joints; a small number are controlled by nearly E‒W faults. Scheelite, occurring on fracture surfaces or at the edges of the quartz vein, is the primary ore mineral alongside pyrite and molybdenite (Supplementary Figs. 2f, g, h, i).

Approximately 900,000 tons of residual diluvial-type W orebodies have been found in Dongyang (Supplementary Fig. 2j). The pegmatite-type W-Mo orebodies are feldspar quartz veins that run through the fault structural belt or the dense jointed belt. The thickness of a single vein is 0.6~0.9 m. Most scheelite occurs as pegmatites and agglomerates in feldspar quartz veins. The molybdenite is small and sparsely disseminated or speckled (Supplementary Figs. 2k, l, m).

**Fluid inclusions**

**Supplementary Table 1.** Samples of fluid inclusions from W-Mo deposits in the mineralization area of Ningshan-Zhen’an.

| No. | Mine area | Sampling position | Mineralization type | Major mineral composition | Host mineral |
| --- | --- | --- | --- | --- | --- |
| QP-1 | Qipangou | 910 level | Quartz-vein type | Qtz+Sch+Bt+Ms | Qtz |
| QP-2 |  |  |  | Qtz+Sch | Qtz |
| QP-3 |  |  |  | Qtz+Sch+Bt+Tr | Sch |
| QP-8 |  | 1130 level | Quartz-vein type | Qtz+Sch | Qtz |
| QP-9 |  |  |  |  | Sch |
| QP-10 |  |  |  |  | Qtz |
| QP-11 |  |  |  |  | Sch |
| QP-12 |  |  |  |  | Sch |
| BgD190816 | Dongyang | Ore block of Hongyatou | Quartz-fluorite-vein type | Qtz+Sch+Fl | Fl |
| BgPM1908-3 |  | Rock mass of Lanbandeng | Pegmatite-dyke type | Qtz+Py+Mo | Qtz |
| BgPM1908-6 |  |  | Quartz-vein type | Qtz+Py | Qtz |
| BgDY-02 |  | Route DY5 after the mineral ministry | Quartz-vein-type | Qtz+Py+Mo | Qtz |
| BgDY-03 |  | Route DY6 after the mineral ministry |  | Qtz+Bt+Phl | Qtz |
| Bg8 | Hetaoping | Section Ⅴ11-12 (4 m) | Quartz-vein-type | Qtz+Py | Qtz |
| BgHT01 |  | Mineral ministry | Beryl-quartz-vein type | Qtz+Mrp+Brl | Qtz |
| Bg56zk01-5 | Yanggou-Di’ergou | 56ZK01 (351 m) | Quartz-fluorite-vein type | Qtz+Fl | Fl |
| BgH04 |  | ZK002 (537.13 m) | Pegmatite in blind rock mass | Qtz | Qtz |
| Bg56ZK01-9 |  | 56ZK01 (665.47 m) |  | Qtz | Qtz |
| BgLPD1ZK06 |  | LPD1ZK06 | Quartz-vein type | Qtz+Mo | Qtz |
| BgD190813  BgD190814-1  BgD190814-3  BgD190814-2 | Guilingou | South of the mineral area  PD6  PD4  Mineral ministry | Quartz-vein type | Qtz+Bt+Phl  Qtz+Ms+Mo  Qtz+Mo  Qtz+Mo | Qtz  Qtz  Qtz  Qtz |
| BgJK-01 | Xiangping of Jiangkou | TC25 | Quartz-vein type | Qtz+Sch | Qtz |

Brl. beryl, Bt. biotite, Fl. Fluorite, Mo. Molybdenite, Mrp. mariposite, Ms. muscovite, Phl. phlogopite, Py. pyrite, Qtz. Quartz, Sch. scheelite.

**
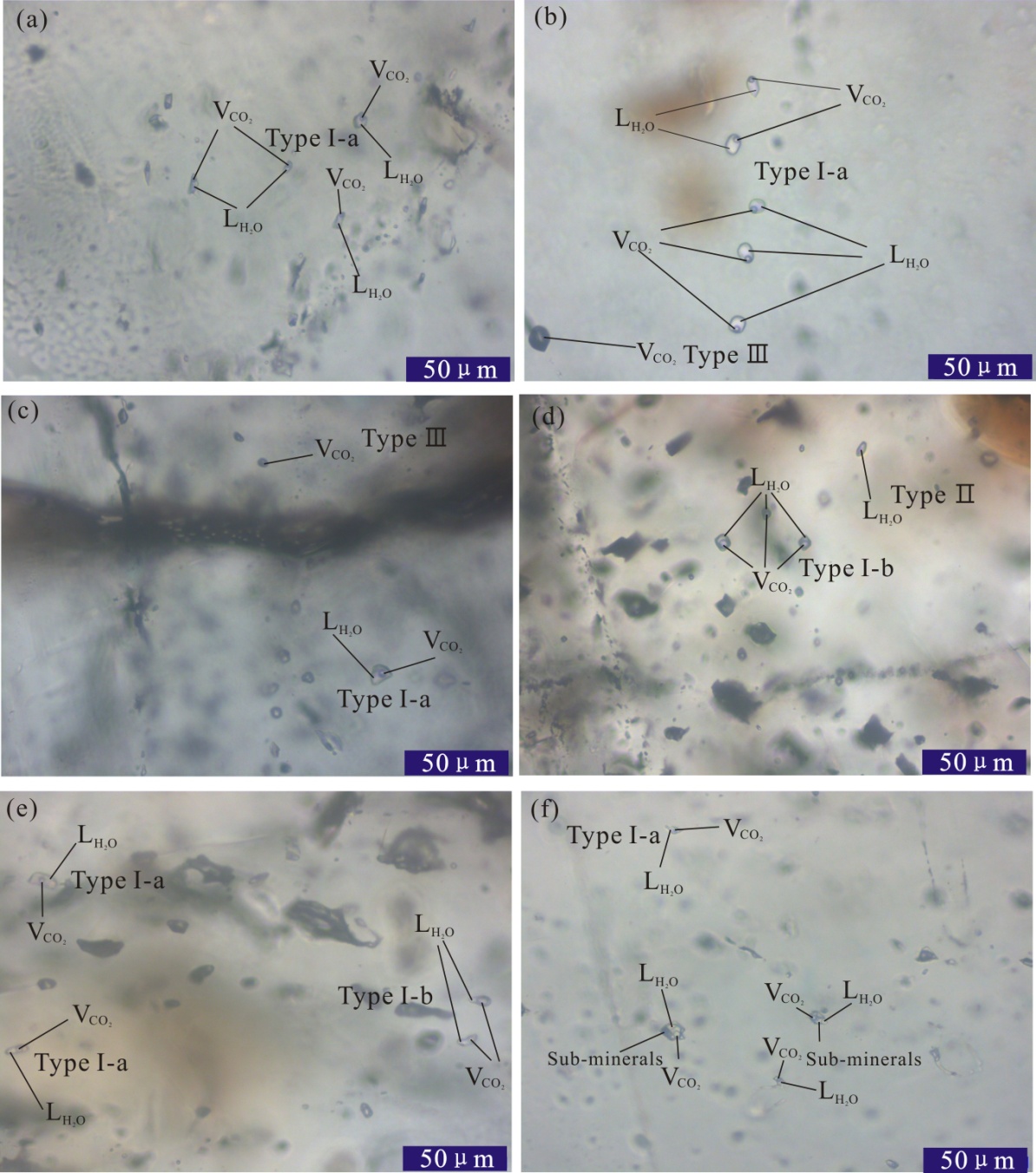
**

**Supplementary Figure 3.** Micropetrographic characteristics of mineral-fluid inclusions of W-Mo deposits in the mineralization area of Ningshan-Zhen'an.

**
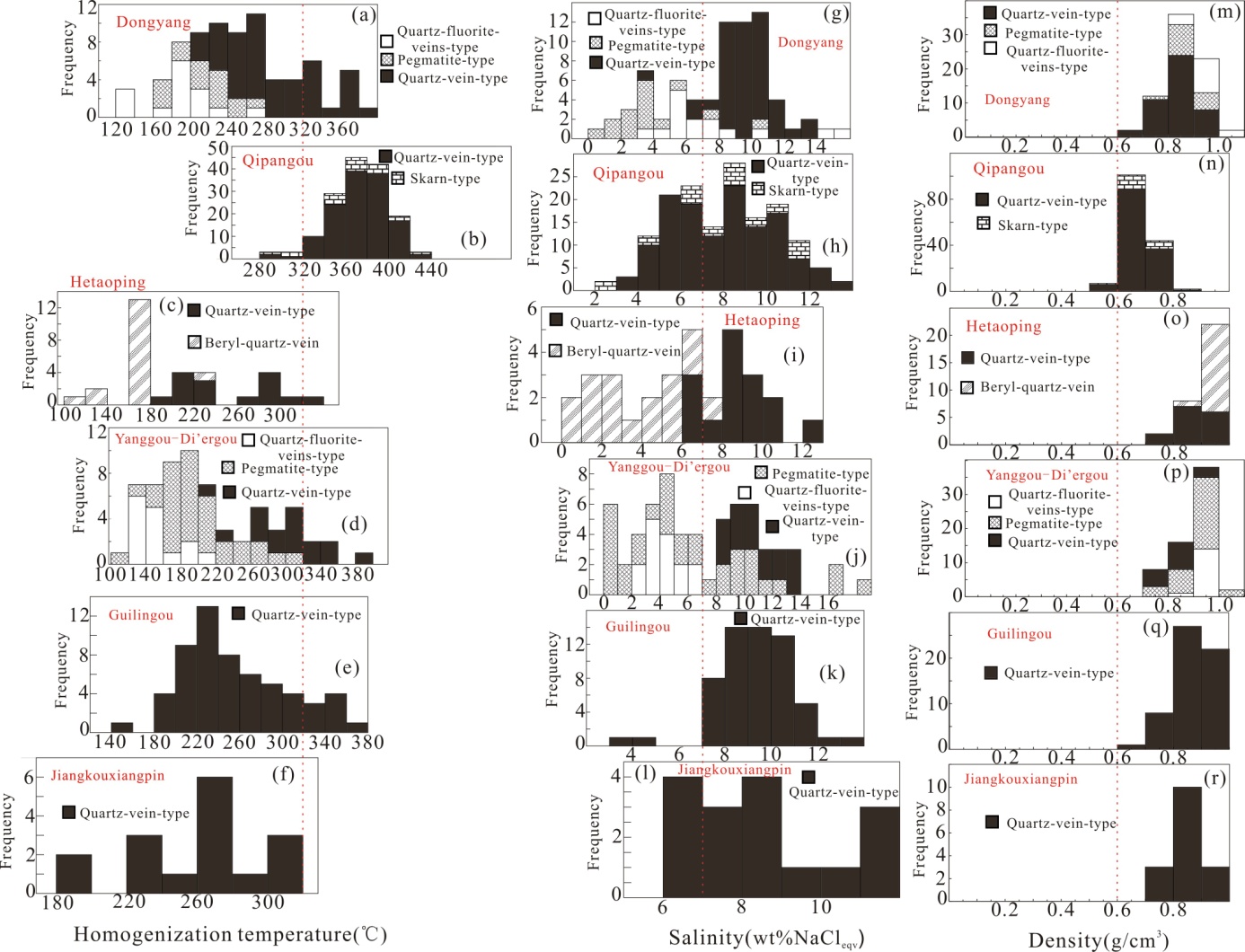
**

**Supplementary Figure 4.** Statistical histogram of the homogenization temperature, salinity and density of fluid inclusions of different W–Mo mineralization types in various mine areas in the mineral concentration zone.

**Supplementary Table 2.** List of the main indices for the fluid inclusions in the W-Mo deposits in the mineralization area of Ningshan-Zhen'an.

| Mine area | No. | Mineralization type | Host mineral | Number | Homogenization temperature (℃) | Freezing point (℃) | Salinity (wt%NaCl_eqv_) | Density (g/cm^3^) | Capture pressure (MPa) | Estimated depth (km) |
| --- | --- | --- | --- | --- | --- | --- | --- | --- | --- | --- |
| Qipangou | QP-1 | Quartz-vein | Quartz | 16 | **371**  (291~424) | **-4.97**  (-8.50~-2.00) | **7.65**  (3.39~12.28) | **0.67**  (0.53~0.78) | **100.83**  (77.84~114.73) | **8.28**  (7.19~8.89) |
|  | QP-2 | Quartz-vein | Quartz | 12 | **355**  (323~383) | **-4.46**  (-6.90~-2.80) | **7.05**  (4.65~10.36) | **0.70**  (0.63~0.76) | **96.41**  (88.53~105.86) | **8.09**  (7.72~8.51) |
|  | QP-3 | Quartz-vein | [Scheelite](https://fanyi.so.com/?src=onebox#scheelite) | 21 | **369**  (284~423) | **-5.24**  (-8.20~-1.20) | **8.08**  (2.07~11.93) | **0.69**  (0.57~0.86) | **100.43**  (78.07~116.87) | **8.26**  (7.20~8.98) |
|  | QP-8 | Quartz-vein | Quartz | 18 | **372**  (323~415) | **-5.63**  (-8.10~-2.10) | **8.63**  (3.55~11.81) | **0.69**  (0.61~0.79) | **101.86**  (86.55~114.42) | **8.33**  (7.62~8.88) |
|  | QP-9 | Quartz-vein- | [Scheelite](https://fanyi.so.com/?src=onebox#scheelite) | 20 | **380**  (319~410) | **-4.74**  (-7.50~-2.10) | **7.42**  (3.55~11.10) | **0.65**  (0.57~0.81) | **103.36**  (88.41~112.84) | **8.40**  (7.71~8.81) |
|  | QP-10 | Quartz-vein | Quartz | 18 | **363**  (295~413) | **-4.16**  (-7.10~-2.60) | **6.62**  (4.34~10.61) | **0.68**  (0.60~0.77) | **98.26**  (78.75~113.82) | **8.17**  (7.24~8.85) |
|  | QP-11 | Quartz-vein | [Scheelite](https://fanyi.so.com/?src=onebox#scheelite) | 33 | **380**  (330~421) | **-6.03**  (-9.80~-3.20) | **9.15**  (5.26~13.72) | **0.68**  (0.61~0.78) | **104.34**  (91.09~116.07) | **8.44**  (7.84~8.95) |
|  | QP-12 | Quartz-vein | [Scheelite](https://fanyi.so.com/?src=onebox#scheelite) | 16 | **379**  (356~397) | **-5.06**  (-7.60~-3.10) | **7.87**  (5.11~11.22) | **0.66**  (0.60~0.73) | **103.40**  (97.81~108.88) | **8.40**  (8.15~8.64) |
| Dongyang | BgD190816 | Quartz-fluorite-vein | Fluorite | 15 | **189**  (133~262) | **-4.90**  (-11.20~-2.10) | **7.49**  (3.55~15.17) | **0.93**  (0.83~1.04) | **51.36**  (36.93~70.42) | **5.48**  (3.69~6.80) |
|  | BgPM1908-3 | Pegmatite | Quartz | 15 | **213**  (165~269) | **-2.31**  (-7.40~-0.50) | **3.77**  (0.88~10.98) | **0.88**  (0.80~0.94) | **54.68**  (39.42~69.80) | **5.82**  (3.94~6.77) |
|  | BgPM1908-6 | Quartz-vein | Quartz | 16 | **263**  (216~324) | **-6.34**  (-9.50~-2.10) | **9.53**  (3.55~13.40) | **0.86**  (0.78~0.93) | **72.35**  (59.44~89.95) | **6.89**  (6.19~7.79) |
|  | BgDY-02 | Quartz-vein | Quartz | 15 | **327**  (210~392) | **-6.51**  (-8.20~-5.20) | **9.83**  (8.14~11.93) | **0.77**  (0.68~0.94) | **90.12**  (58.18~108.51) | **7.77**  (6.12~8.63) |
|  | BgDY-03 | Quartz-vein | Quartz | 14 | **268**  (206~310) | **-6.26**  (-7.20~-4.90) | **9.53**  (7.73~10.73) | **0.86**  (0.78~0.93) | **73.80**  (56.81~85.03) | **6.97**  (6.04~7.55) |
|  | 1 | Skarn | [Scheelite](https://fanyi.so.com/?src=onebox#scheelite) | 26 | **258**  (209~283) | -8.2~-3.6 | **6.68**  (5.41~11.93) | 0.81~0.95 | Liu, 2013^13^ | |
|  | 2 | Quartz-vein | Quartz | 16 | **186**  (139~192) | -5.6~-3.2 | **6.25**  (4.34~8.68) | 0.71~0.89 |  |  |
|  | 3 | Skarn | Quartz | 21 | **264**  (227~319) | -8.2~-3.0 | **7.12**  (4.96~11.93) | 0.74~0.93 |  |  |
|  | 4 | Quartz-fluorite-vein | Fluorite | 26 | **179**  (142~185) | -4.3~-1.1 | **5.76**  (1.91~6.88) | 0.80~0.87 |  |  |
|  | 5 | Quartz-vein | Quartz | 35 | **246**  (187~296) | -8.3~-1.9 | **8.42**  (3.32~12.05) | 0.78~0.92 |  |  |
| Hetaoping | Bg8 | Quartz-vein | Quartz | 15 | **253**  (198~328) | **-5.65**  (-8.40~-3.90) | **8.70**  (6.30~12.16) | **0.87**  (0.74~0.95) | **69.35**  (54.72~89.16) | **6.72**  (5.92~7.75) |
|  | BgHT01 | Beryl-quartz-vein | Quartz | 17 | **167**  (116~237) | **-2.18**  (-4.40~-0.10) | **3.60**  (0.18~7.02) | **0.93**  (0.88~0.96) | **41.81**  (14.82~64.67) | **4.78**  (1.48~6.49) |
| Yanggou-Di’ergou | Bg56zk01-5 | Quartz-fluorite-vein | Fluorite | 15 | **154**  (123~208) | **-2.60**  (-4.20~-1.60) | **4.31**  (2.74~6.74) | **0.95**  (0.88~0.97) | **40.62**  (32.15~53.62) | **4.23**  (3.21~5.85) |
|  | BgH04 | Pegmatite | Quartz | 18 | **197**  (147~282) | **-2.71**  (-7.90~-0.10) | **4.26**  (0.18~11.58) | **0.90**  (0.75~0.97) | **46.67**  (20.84~62.31) | **5.09**  (2.08~6.36) |
|  | Bg56ZK01-9 | Pegmatite | Quartz | 15 | **202**  (117~303) | **-6.39**  (-14.80~-0.30) | **9.20**  (0.53~18.47) | **0.93**  (0.70~1.02) | **53.61**  (30.79~73.98) | **5.67**  (3.08~6.99) |
|  | BgLPD1ZK06 | Quartz-vein | Quartz | 16 | **303**  (215~383) | **-7.31**  (-9.60~-5.40) | **10.81**  (8.41~13.51) | **0.82**  (0.72~0.94) | **83.84**  (59.67~106.10) | **7.47**  (6.21~8.52) |
| Guilingou | BgD190813 | Quartz-vein | Quartz | 11 | **296**  (227~370) | **-5.93**  (-7.50~-3.00) | **9.07**  (4.96~11.10) | **0.81**  (0.69~0.89) | **81.49**  (60.78~101.62) | **7.34**  (6.27~8.33) |
|  | BgD190814-1 | Quartz-vein | Quartz | 15 | **249**  (208~324) | **-6.49**  (-8.80~-4.90) | **9.80**  (7.73~12.62) | **0.88**  (0.76~0.94) | **68.60**  (57.56~88.61) | **6.69**  (6.08~7.72) |
|  | BgD190814-2 | Quartz-vein | Quartz | 15 | **285**  (221~327) | **-6.28**  (-9.20~-2.10) | **9.48**  (3.55~13.07) | **0.83**  (0.78~0.93) | **78.37**  (61.10~90.51) | **7.20**  (6.29~7.81) |
|  | BgD190814-3 | Quartz-vein | Quartz | 17 | **209**  (159~240) | **-5.92**  (-7.90~-4.40) | **9.07**  (7.02~11.58) | **0.92**  (0.89~0.97) | **57.59**  (43.34~66.04) | **6.08**  (5.22~6.56) |
| Jiangkouxiangping | BgJK-01 | Quartz-vein | Quartz | 16 | **259**  (185~318) | **-5.56**  (-8.00~-4.00) | **8.58**  (6.45~11.70) | **0.86**  (0.77~0.95) | **71.06**  (50.81~87.22) | **6.82**  (5.68~7.66) |

Note: The numbers in bold are the mean values, and the numbers in parentheses are the ranges of the values.

**
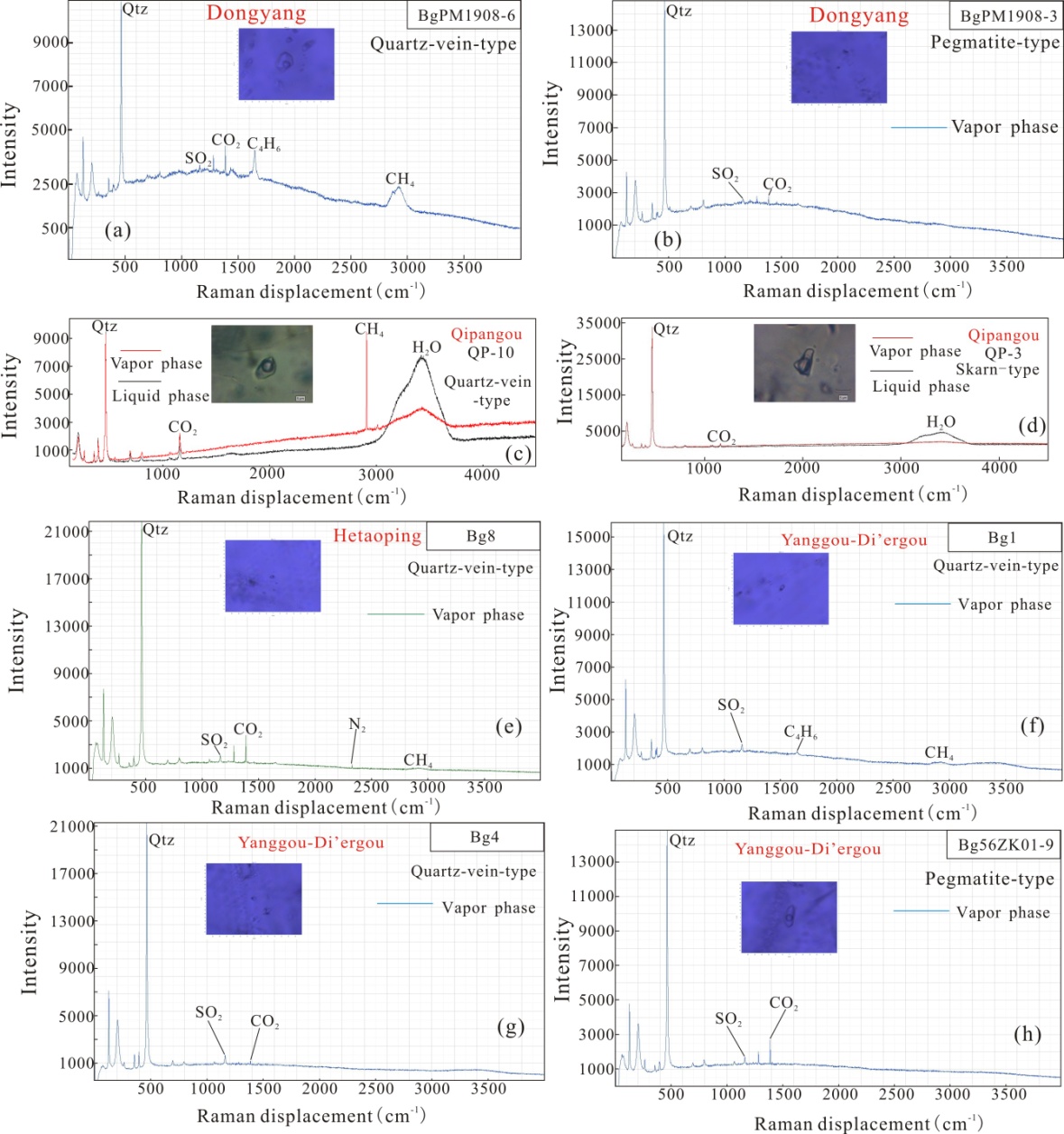
**

**Supplementary Figure 5.** Laser Raman spectra of fluid inclusions in W-Mo deposits in the mineralization area of Ningshan-Zhen'an.

**Stable isotope**

**Supplementary Table 3.** H-O isotopic compositions of W-Mo deposits in the mineralization area of Ningshan-Zhen'an.

| Mine area | No. | Sample | Mineral | δD_H2O_ (‰) | δ^18^O_Quartz_(‰) | δ^18^O_H2O_ (‰) | Homogenization temperature (℃) |
| --- | --- | --- | --- | --- | --- | --- | --- |
| Dongyang | D190816 | Ore-bearing quartz-fluorite vein | Quartz | -74.8 | 12.2 | -0.20 | 189 |
|  | PM1908-6 | Ore-bearing quartz vein | Quartz | -77.5 | 10.8 | 2.45 | 263 |
| Qipangou | QP-1 | Ore-bearing quartz vein | Quartz | -71.0 | 11.0 | 6.26 | 371 |
|  | QP-2 | Ore-bearing quartz vein | Quartz | -69.8 | 11.6 | 6.42 | 355 |
|  | QP-3 | Ore-bearing skarn | Quartz | -64.9 | 10.9 | 6.09 | 369 |
| Hetaoping | HT01 | Beryl-quartz veins | Quartz | -78.0 | 12.6 | -1.49 | 167 |
| Yanggou-Di’ergou | LPD1ZK06 | Ore-bearing quartz vein | Quartz | -80.1 | 10.5 | 3.72 | 303 |
| Guilingou | D190814-1 | Ore-bearing pegmatite | Quartz | -78.1 | 7.3 | -1.71 | 249 |
|  | D190814-2 | Ore-bearing quartz vein | Quartz | -77.6 | 12.2 | 4.75 | 285 |
|  | D190814-3 | Ore-bearing quartz vein | Quartz | -73.6 | 11.2 | 0.08 | 209 |
| Jiangkouxiangping | JK-1 | Ore-bearing quartz vein | Quartz | -69.7 | 11.5 | 2.96 | 259 |
| Dongyang | HYT-2 | Skarn-type | Sericite | -75.9 | 13.8 | 11.4 | 260 |
|  | HYT-2 |  | Sericite | -76.4 | 12.0 | 9.6 | 260 |

Note: The data on skarn-type W mineralization in Dongyang are from Liu, 2013^13^.

**Supplementary Table 4.** S-isotopic compositions of W-Mo deposits in the mineralization area of Ningshan-Zhen'an.

| Mine area | No. | Sample | Mineral | δ^34^S/‰ |
| --- | --- | --- | --- | --- |
| Qipangou | QP-1 | Ore-bearing quartz vein | Pyrite | 10.0 |
|  | QP-2 | Ore-bearing quartz vein | Pyrite | 10.2 |
|  | QP-3 | Ore-bearing quartz vein | Pyrite | 8.8 |
| Yueheping | D190830 | Ore-bearing skarn | [Molybdenite](https://fanyi.so.com/#molybdenite) | 6.1 |
| Guilingou | D190814-1 | Ore-bearing pegmatite | [Molybdenite](https://fanyi.so.com/#molybdenite) | 4.0 |
|  | D190814-2 | Ore-bearing quartz vein | [Molybdenite](https://fanyi.so.com/#molybdenite) | 3.6 |
|  | D190814-3 | Ore-bearing quartz vein | [Molybdenite](https://fanyi.so.com/#molybdenite) | 4.3 |
| Daxigou | D190819 | Ore-bearing granite porphyry | [Molybdenite](https://fanyi.so.com/#molybdenite) | 0.1 |
| Yanggou-Di’ergou | LPD1ZK06 | Ore-bearing quartz vein | [Molybdenite](https://fanyi.so.com/#molybdenite) | 7.1 |
